# Towards a Long-Chain Perfluoroalkyl Replacement: Water and Oil Repellent Perfluoropolyether-Based Polyurethane Oligomers

**DOI:** 10.3390/polym13071128

**Published:** 2021-04-02

**Authors:** Liying Wei, Tugba D. Caliskan, Philip J. Brown, Igor Luzinov

**Affiliations:** 1 Department of Materials Science and Engineering, Clemson University, Clemson, SC 29634, USA; liying@g.clemson.edu (L.W.); tdemir@g.clemson.edu (T.D.C.); pjb@clemson.edu (P.J.B.); 2 Department of Chemical Engineering, Faculty of Engineering, Ankara University, 06100 Tandogan, Turkey

**Keywords:** oil repellency, water repellency, perfluoropolyethers, oligomer, fluorinated polyurethanes, oleophobicity, hydrophobicity

## Abstract

Original perfluoropolyether (PFPE)-based oligomeric polyurethanes (FOPUs) with different macromolecular architecture were synthesized (in one step) as low-surface-energy materials. It is demonstrated that the oligomers, especially the ones terminated with CF_3_ moieties, can be employed as safer replacements to long-chain perfluoroalkyl substances/additives. The FOPU macromolecules, when added to an engineering thermoplastic (polyethylene terephthalate, PET) film, readily migrate to the film surface and bring significant water and oil repellency to the thermoplastic boundary. The best performing FOPU/PET films have reached the level of oil wettability and surface energy significantly lower than that of polytetrafluoroethylene, a fully perfluorinated polymer. Specifically, the highest level of the repellency is observed with an oligomeric additive, which was made using aromatic diisocyanate as a comonomer and has CF_3_ end-group. This semicrystalline oligomer has a glass transition temperature (*T*_g_) well above room temperature, and we associate the superiority of the material in achieving low water and oil wettability with its ability to effectively retain CF_3_ and CF_2_ moieties in contact with the test wetting liquids.

## 1. Introduction

This work focuses on attaining water and oil repellency of engineering thermoplastics, such as polyethylene terephthalate (PET), with the addition of perfluoropolyethers (PFPEs)-based oligomeric polyurethanes. Repellency has been one of the critical targets in designing practical polymer-based materials contacting with aqueous and/or oily liquid media [1,2,3]. To this end, a number of engineering thermoplastics have low wettability by water and demonstrate significant water repellency [4,5,6,7]. In contrast to hydrophobic polymers, only fluorinated polymers demonstrate some level of oil repellency [4,8,9,10,11,12]. However, with a few exceptions (e.g., polytetrafluoroethylene and polyvinylidenefluoride), the higher cost of these polymers and/or their mechanical properties generally prevents their widespread applications as engineering materials.

For decades, long-chain perfluoroalkyl (LCPFAs, C_n_F_2n+1_, n ≥ 7) containing chemicals have been widely used as additives or (co)monomers to obtain materials with low levels of oil wettability [2,3,13,14]. However, LCPFAs (sometimes referred to as “forever chemicals” [15] in popular media) have been phased out of industrial applications and production due to their persistency in the environment and toxicological/bioaccumulative impact on humans and wildlife [3,16,17,18]. In this respect, this paper concentrates on enhancing hydrophobicity and oleophobicity of engineering thermoplastics via the addition of PFPE-based polyurethane oligomers, which do not contain LCPFAs. PFPEs are macromolecules possessing –CF_2_–, –CF_2_–CF_2_–, and –CF(CF_3_) –CF_2_– molecular fragments in their backbone that are separated by oxygen atoms. Currently, PFPEs are considered as potentially safer replacements for LCPFAs [3,19,20,21]. PFPEs have numerous advantages, such as high chemical inertness and radiation resistance, low surface tension (20–22 mN/m) [22], nonflammability, low toxicity, optical transparency, and low volatility [22,23,24]. However, as pure materials, they cannot serve as effective water/oil repellent additives for engineering thermoplastics because of their immiscibility and incompatibility with polymer matrices [22,25].

To this end, perfluoropolyether derived (co)polymers and cross-linked materials have been shown in our previous works and that of others to have the ability to serve as hydrophobic/lyophobic materials and interfaces [20,21,22,23,25,26,27,28,29,30,31,32,33,34,35,36,37]. In particular, we have found that when PFPE-based triblock polyesters [20,21,37] or methacrylic molecular brushes [36] are added to engineering thermoplastic (PET, nylon 6, or polymethyl methacrylate) films, they readily migrate to the film surface, imparting significant water and oil repellency to the thermoplastic boundary. Specifically, the macromolecular additives populated the boundary with PFPE segments terminated with C_4_F_9_-perfluoroalkyl moiety, which cannot yield unsafe long-chain perfluoroalkyl carboxylic acids. The lowest wettability was demonstrated by the additives, where a significant mismatch in the affinity between the C_4_F_9_-PFPE segments and host matrix (preferring interaction with other parts of the macromolecules) promoted stretching and densification of the PFPE segments delivering the low surface energy C_4_F_9_- functionality to the material boundary.

While the additives reported by us were remarkably effective in delivering the low surface energy CF_3_ (γ ≈ 6 mN/m) [9,10] and CF_2_ (γ ≈ 18 mN/m) [9,10] moieties to the material surface, it was necessary to employ an elaborated multistep synthetic procedure to obtain the macromolecules (molecular brushes and triblock polyesters). With this in mind, we now report on the synthesis, properties, and wettability of the original PFPE-based polyurethane oligomers (FOPUs) designed to serve as (easy-to-make in one step) low-surface energy non-LCPFA additives to impart water and oil repellency to engineering thermoplastics. We targeted the synthesis of lower molecular weight macromolecules to ensure their better compatibility with host matrices and potentially having higher rates of diffusion and, therefore, migrating more readily to the material boundary. The synthesis of low-surface energy fluorinated polyurethanes (many of them are produced commercially) is well established and allows manipulation of their structure and properties in a wide range [30,38,39,40,41,42,43,44,45,46,47,48,49,50,51,52,53,54,55,56,57].

Four different FOPUs with and without C_4_F_9_-PFPE end-segments were obtained and contrasted. To vary the properties of the materials, we used two different (alkyl and aromatic) diisocyanate comonomers. FOPUs were solvent-blended with PET to access their efficiency as water/oil repellent additives to the engineering thermoplastic. In general, the obtained blended films demonstrated low wettability with water and hexadecane depending on the oligomer composition, confirming the efficiency of our polymer modification strategy. We expect that this surface modification method can be readily transferred to a number of other essential thermoplastic polymers. 

## 2. Results and Discussion 

### 2.1. Synthesis and Characterization of FOPUs

**Figure 1:**
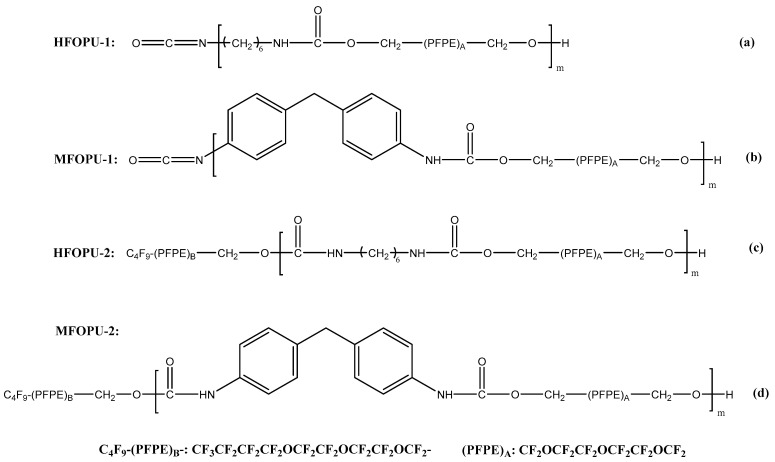
Chemical structure of (**a**) HFOPU-1 polyurethane, (**b**) MFOPU-1 polyurethane, (**c**) HFOPU-2 polyurethane, and (**d**) MFOPU-2 polyurethane.

Figure 1 depicts the chemical structure of FOPUs. Four distinct polyurethanes were synthesized from two different diisocyanates (alkyl-based 1, 6 hexamethylene diisocyanate, HDI and aromatic-based 4,4′-methylenebis(phenyl isocyanate), MDI) via step-growth polymerization. As the comonomer and end-segment fluorinated ether alcohols 1H, 1H, 11H, 11H- fluorinated-3,6,9-trioxaundecane-1,11-diol (PFPE-diol) and 1H, 1H-fluorinated-3,6,9-trioxatridecan-1-ol (C_4_F_9_-PFPE-OH) were used, respectively. The materials and experimental details for FOPUs synthesis and structural characterization are provided in Supporting Information (SI: S1–S3). Attenuated total reflectance Fourier transform infrared spectroscopy (ATR-FTIR) and 19F nuclear magnetic resonance (NMR) were performed to confirm the chemical structure of the obtained FOPUs. In general, the IR and NMR results (SI: S3, Figures S2–S4, and Table S1) indicated that targeted FOPUs were obtained by the synthetic procedure employed. The molecular weight and polydispersity index (PDI) for FOPUs were determined by GPC analysis (SI: Table S2). The data obtained revealed that FOPU oligomers with weight average molecular weight (*M*_w_) between 2500–4000 g/mol and PDI between 1.2–1.8 were obtained. Based on the oligomer structure, we estimated that the number of repeating units for HFOPU-1, MFOPU-1, HFOPU-2, and MFOPU-2 chain was ~5, 4, 6, and 3, respectively. Based on the structure of end-groups, *M*_w_, and number of repeating units for HFOPU-1, MFOPU-1, HFOPU-2, and MFOPU-2 macromolecules, we estimated that the atomic concentration of fluorine in the oligomeric chains is quite close and is about 23% for HFOPU-1, 21% for MFOPU-1, 25% for HFOPU-2, and 25% for MFOUPU-2. It is necessary to point out that during the storage at ambient conditions, the isocyanate end-group of HFOPU-1 and MFOPU-1 can react with water present in the air. As a result, the end-group can be transformed into amino group, as reported elsewhere [58,59]. This possible transformation was not investigated here. However, we suggest that this process cannot significantly alter the material’s surface properties, since one high surface energy polar isocyanate group is replaced with another high surface energy amino group.

Thermogravimetric analysis, TGA showed (SI: Figure S5) that the monomers are entirely consumed during the polycondensation. Though PU fractions of lower molecular weight (with the thermal stability between 160 °C and 200 °C) is present in FOPUs, the major fraction (>80%) of the obtained oligomers exhibits a decomposition temperature (*T*_d_) between 230 and 320 °C, indicating significant thermal stability of the higher molecular weight materials. DSC (differential scanning calorimetry) analysis was used to determine the glass transition temperature (*T*_g_) and melting temperature (*T*_m_) for the oligomers (SI: Figure S6 and Table S2). The results indicate that FOPUs have both *T*_g_ and *T*_m_; therefore, they are semicrystalline materials with a degree of crystallinity on the level of 30–35% (SI: S4 and Table S2). It is necessary to point that the presence of C_4_F_9_-PFPE-end-segment in the polyurethane structure does not significantly influence the thermal transitions. The midpoint *T*_g_ for HFOPUs is ~−30 °C, while *T*_m_ (at maximum) is 60–64 °C. The thermal transitions for MFOPUs are occurring at significantly higher temperatures. Specifically, the midpoint *T*_g_ is approximately 45 °C and *T*_m_ is ~125 °C. We connect these differences with the chemical structure of oligomers. HFOPUs possess more flexible aliphatic polyurethane segments in the backbone, while the presence of the rigid phenyl rings in MFOPUs increases their thermal transition temperatures [7,60,61]. 

### 2.2. Wettability of FOPUs

We evaluated the extent of water and oil repellency of the annealed pure FOPU films using static contact angles of water (WCA) and hexadecane (HDCA), respectively. FOPU films were prepared by dip coating, dried at ambient conditions for 16 h, and then annealed at 140 °C for 3 h under vacuum. The annealing temperature was selected to be above the FOPUs’ thermal transitions. Prior to the contact angle measurement, we evaluated the solubility of the oligomers in the wetting liquids and found out that FOPUs are not soluble in water and hexadecane. In addition to CA measurements, the surface energy (σ) of FOPUs was also estimated from HDCA and WCA data using the Owens–Wendt method (SI: S7) [62]. The measured WCA, HCA, and surface energy values for annealed pure FOPU films are presented in Table 1. One can see that the materials possess significant levels of water and oil repellency. The repellency is on par with or exceeding that of fully fluorinated polytetrafluoroethylene (PTFE, TEFLON). In fact, under our experimental conditions, we measured WCA and HCA for PTFE as 118° and 51°, respectively, which correlated well with the values reported in the scientific literature [63,64]. The PTFE surface energy of 17.5 mN/m, calculated by the Owens–Wendt method from our experimentally measured contact angles, was also close to the one typically reported [9,10].

In general, materials containing C_4_F_9_-PFPE- end-segments showed significantly lower wettability and surface energy than the oligomers having the same polyurethane chain, which was not terminated with the perfluoroalkyl short moiety. This superiority of materials containing C_4_F_9_– groups can be expected, since the films’ surface is always preferentially occupied by the fragments of the molecular chains with the lowest surface energy [7]. Indeed, CF_3_– groups possess the lowest surface energy of ~6 mN/m. Therefore, the oligomers with perfluoroalkyl end-groups showed higher CA values.

#### 2.2.1. Water and Oil Repellency of HFOPU-1 and MFOPU-1

We note that HFOPU oligomers containing aliphatic PU chain demonstrated somewhat lower oil and water repellency than MFOPU having aromatic groups in the polyurethane backbone. The difference could be connected to the surface energy of the repeating units constituting macromolecular chains. To this end, we estimated surface energies and their (polar and dispersive) components (SI: S6) for the molecular segments constituting FOPUs using algorithms reported elsewhere [20,36]. The calculated values are presented in SI, Table S4. One can see that the HFOPU repeating unit has lower surface energy than MFOPU and, in principle, has to demonstrate lower wettability by water and HD. The opposite behavior observed is connected to specific conformations of the FOPUs chains located at the material surface. Specifically, MFOPU chains expose a significantly higher number of low surface energy fluorinated groups (-CF_2_-) to the material boundary. It is especially evident from the analysis of HDCA, since HD has much lower surface energy (26.4 mN/m [65]) than water (72 mN/m [5]) and, thus, is more sensitive to the presence of the low surface energy fluorinated groups on the surface. In general, it was shown that liquids with bulky molecules like hexadecane are rather suitable for contact angle measurements to characterize energetics of fluorinated polymer surfaces [66,67].

The classical Young’s equation allows simple estimation of a solid surface wettability by a liquid [5]:

(1) $$\cos\theta =(\gamma_{1}-\gamma_{12})/\gamma_{2}$$

where *γ*_1_, *γ*_2_, and *γ*_12_ represent the surface/interfacial tension at solid–vapor, liquid–vapor, and solid–liquid boundaries. It is evident that for material to demonstrate a 60° contact angle with hexadecane, having a surface energy of 26.4 mN/m, its surface energy less liquid–surface interfacial tension has to be on the level of 13.2 mN/m. In the case of hydrocarbon oils that have polar contributions to the surface tension equal to zero [5,65], the interfacial tension can be estimated via the following equation [68,69]:

(2) $$\gamma_{12}=\gamma_{1}+\gamma_{2}-\frac{4\gamma_{1}^{d}\gamma_{2}^{d}}{\gamma_{1}^{d}+\gamma_{2}^{d}}$$

where the d subscripts refer to the dispersive contributions to the surface tensions *γ*. Table S4 (SI) shows, estimated by us, surface energy and its components for PFPE segments present in both HFOPU and MFOPU backbones. We can approximate that interfacial tension at PFPE/HD contact is about 9.5 mN/m and calculated HDCA is, therefore, about ~43 degrees. This result indicated that the surface of HFOPU-1 and MFOPU-1 is occupied by low surface energy –CF_2_– groups, which are typically reported to have a surface energy of ~18 mN/m. This value was determined in classical works by Zisman et al. using homologous series of n-alkanes as wetting liquids and polytetrafluoroethylene consisting of –CF_2_–CF_2_- monomeric units as a substrate [8,10]. Specifically, *γ*_1_ = 18.5 mN/m was measured for PTFE by Zisman with coworkers. Dispersive contributions to the surface tension of PTFE (*γ*_d_) is about 17.0 mN/m (as calculated from the fraction of dispersive contribution to γ_1_ that is equal to 0.92 for PTFE [5]). Interfacial tension at PTFE/HD contact calculated by Equation (2) is then about 3.5 mN/m and has HDCA estimated by Equation (1) to be ~55 degrees. 

**Figure 2:**
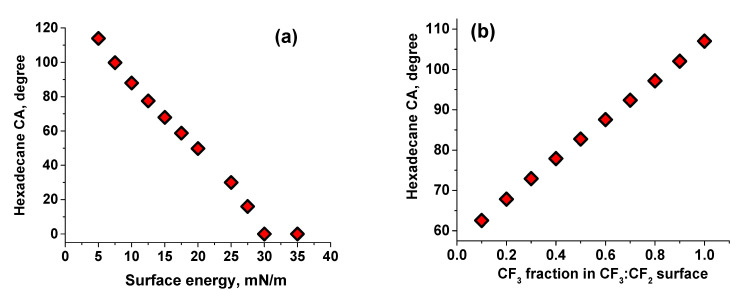
(**a**) HDCA vs. surface energy for a range of hypothetical polymeric surfaces with a fraction of dispersive contribution to *γ*_1_ on the same level as PTFE (0.92). (**b**) HDCA dependence on the composition of surface occupied by a mixture of CF_3_ and CF_2_ functionalities.

Figure 2a shows HDCA versus surface energy for a range of hypothetical polymeric surfaces with a fraction of dispersive contribution to γ_1_ on the same level as PTFE (0.92) calculated using Equations (1) and (2). Based on this graph, HFOPU-1 has a surface energy of ~17.8 mN/m, while MFOPU-1 has a surface energy of ~15.9 mN/m (Table 1). The surface energy values estimated from the contact angle of hexadecane are close, but to some extent different from the ones obtained by the Owens–Wendt method utilizing both WCA and HDCA. We associate this difference with the differences in the size of the wetting liquids, which plays a significant role in the wettability of polymer surfaces [20,21,36]. Based on molecular weight and chemical structure, the size of water molecule is about an order of magnitude smaller than that of hexadecane. Specifically, the molecular volumes for water and hexadecane at 20 °C are 30 and 458 Å^3^, respectively [70]. Therefore, water can penetrate to a greater degree into the layer of fluorinated polyurethanes and contact with more moieties compared to hexadecane.

From values of HD contact angle, we can suggest that hexadecane contacting only -CF_2_– groups on the surface of HFOPU-1/MFOPU-1 and that effective surface energy of the groups in the PFPE based polyurethanes is lower than the one in PTFE. It also appears that the effective energy of –CF_2_– groups in the aromatic-based FOPU is lower than that in the aliphatic-based material. We associate this phenomenon with higher density (and lower mobility) of the amorphous part of MFOPU-1 material, which is (in contrast to HFOPU-1) well below *T*_g_ at ambient temperature. Indeed, according to the free volume theory of glass transition and experimental observations, *T*_g_ is the iso-free-volume state of macromolecular materials, where all materials have approximately the same fractional free volume at the transition [7]. The volume coefficient of thermal expansion of polymers is about two times higher above *T*_g_ than the one below *T*_g_. Obviously, the density of HFOPU-1, which is 55 °C above *T*_g_, is significantly lower than the density of MFOPU-1. The packing density increase with an associated decrease in mobility of the fluorinated moieties results in surface immobilization of –CF_2_– groups. Note that the interfacial tension between –CF_2_– groups and HD (estimated by Equations (1) and (2)) is ~3.7 mN/m, while interfacial tension between the adjacent –CH_2_– groups and HD is only ~0.4 mN/m (SI: S6). Due to the thermodynamic condition of surface/interfacial energy minimization, mobile –CF_2_– groups are reorienting in an attempt to avoid contact with the wetting oil that prefer contacts with –CH_2_– groups. Hence, we suggest that the restricted mobility of the low surface energy –CF_2_– group is a key parameter causing higher oleophobicity of MFOPU-1.

#### 2.2.2. Water and Oil Repellency of HFOPU-2 and MFOPU-2

When C_4_F_9_-PFPE- end-segments are incorporated into the FOPU macromolecules, glassy/semicrystalline MFOPU-2 material has lower WCA, HDCA, and surface energy than the rubbery/semicrystalline aliphatic HFOPU-2 (Table 1). As noted above, the higher level of water and oil repellency is connected to the lower mobility of the MFOPU backbone in the amorphous phase that supports effective localization of not only CF_2_, but also CF_3_ moieties at the material surface. In fact, the interfacial tension between CF_3_ and HD is ~14 mN/m ((SI: S6)), and CF_3_/HD contact is even more thermodynamically unfavorable than the CF_2_/HD one. Based on Figure 2a, the surface energy of HFOPU-2 and MFOPU-2 calculated from HDCA are 15 mN/m and 13.1 mN/m, respectively. The decrease in surface energy can only be associated with lower surface energy (~6 mN/m) of CF_3_ moieties [9,10]. Indeed, significant fractions of the surface have to be occupied by CF_3_ terminal groups that have to be in contact with the wetting oil to obtain high contact angles.

To comprehend and visualize the situation, we show in Figure 2b HDCA dependence on the composition of surface occupied by a mixture of CF_3_ and CF_2_ functionalities. To populate the graph Israelachvili and Gee equation for the contact angle on chemically heterogeneous surfaces was employed [71,72,73]:

(3) $${\left(1+\cos\theta \right)}^{2}=f_{1}{\left(1+\cos\theta_{1}\right)}^{2}+f_{2}{\left(1+\cos\theta_{2}\right)}^{2}$$

where *θ* is a contact angle of a liquid on a heterogeneous surface composed of *f*_1_ and *f*_2_ fraction of chemical groups type 1 and type 2, where *θ*_1_ and *θ*_2_ are the HD contact angles on the pure homogeneous surface 1 and 2, respectively. The angles were estimated from Figure 2a using typically reported values for CF_3_ (~6 mN/m) and CF_2_ (~18 mN/m) surface energies. Figure 2b shows that CF_3_:CF_2_ ratio for HFOPU-2 and MFOPU-2 is about 0.26:0.74 and 0.42:0.58, respectively. It is necessary to point that CF_3_ groups (with a molar weight of 39 g/mol) constitute only 1–2.5 wt% of the fluorinated polyurethanes. However, their surface localization is more than an order of magnitude higher than this value.

### 2.3. Morphology of FOPU/PET Films

**Figure 3:**
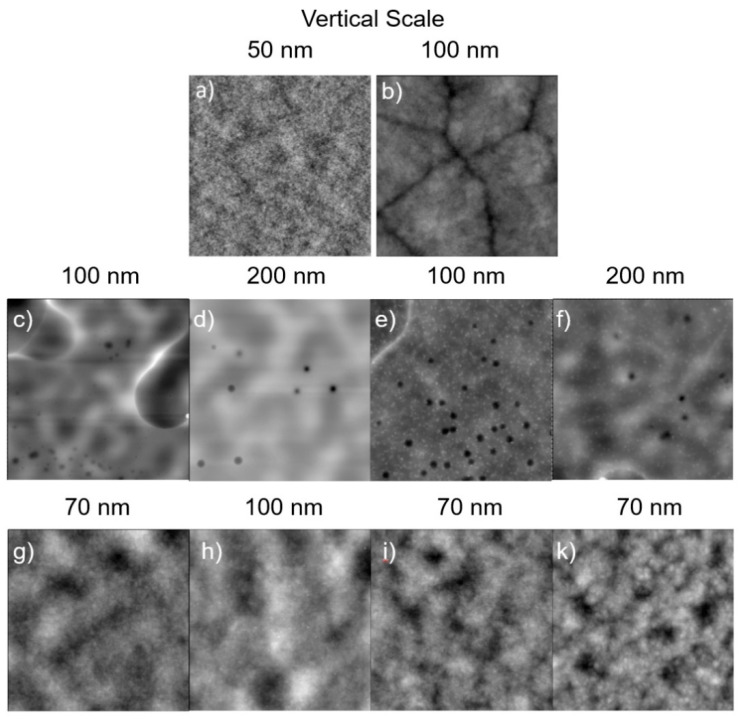
AFM (10 µm × 10 µm) topographical images of polymer films before (**a**,**c**–**f**) and after (**b**,**g**–**k**) annealing. Pure PET (**a**,**b**), and 5% FOPU/PET films (**c**–**k**). Before annealing: (**a**) Pure PET (RMS = 0.3 nm), (**c**) HFOPU-1/PET (RMS = 13.5 nm), (**d**) HFOPU-2/PET (RMS = 15.0 nm), (**e**) MFOPU-1/PET (RMS = 8.0 nm), and (**f**) MFOPU-2/PET (RMS = 17.0 nm). After annealing: (**b**) Pure PET (RMS = 8.0 nm), (**g**) HFOPU-1/PET (RMS = 9.0 nm), (**h**) HFOPU-2/PET (RMS = 16.0 nm), (**i**) MFOPU-1/PET (RMS = 8.5 nm), and (**k**) MFOPU-2/PET (RMS = 11.0 nm). RMS is the root-mean-square roughness determined using AFM software from the topographical images.

PET films blended with 5 wt% of FOPU materials were prepared by dip coating, dried at ambient conditions for 16 h, and then annealed at 140 °C for 3 h under vacuum. The annealing temperature was above the FOPUs’ thermal transitions and the *T*_g_ of PET (70–80 °C [7,60,61]), yet below the melting temperature of PET (250–260 °C [7,60,61]). The micro/nanoscale morphology of the blended films along with the film made from pure PET was visualized using atomic force microscopy (AFM) topographical imaging (Figure 3). It was observed that the films fabricated from solutions are without visible crystal formation. It was also apparent that PET and FOPU oligomers are, to some extent, immiscible and appear to be phase-separated on the AFM topographical images. The annealing significantly influenced the surface morphology of FOPU/PET and PET films. PET crystalline structures are visible on the surface of pure PET films after the thermal treatment. We also noted that, for the annealed FOPU/PET films, PET crystalline structures and phase separation are not clearly observed on the topographical images. It appears that FOPUs spread over the PET surface, forming a continuous layer as a lower surface energy component for thermodynamical reasons [7]. 

**Figure 4:**
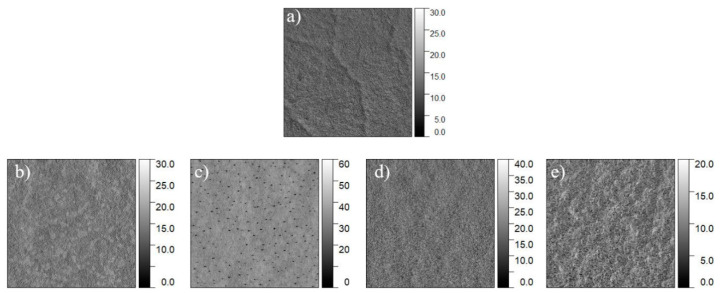
AFM (10 µm × 10 µm) phase images of annealed polymer films of pure PET (**a**) and 5 wt% FOPU/PET films (**b**–**e**). (**b**) HFOPU-1/PET, (**c**) HFOPU-2/PET, (**d**) MFOPU-1/PET, and (**e**) MFOPU-2/PET films. The scale is in degree.

AFM phase images (Figure 4) were employed to clarify this matter, since they are particularly sensitive to heterogeneity in surface composition [20,74]. The phase images did not show the top surface layer as discontinuous and only partially covering the film surface. This indicates that, upon the thermal treatment, FOPU spreads over the PET surface and forms a continuous layer. Namely, the entire surface of the FOPU/PET films is covered with nanoscale fluorinated polyurethane oligomer layers.

### 2.4. Wettability of FOPU/PET Films

We evaluated the extent of water and oil repellency of the annealed FOPU/PET films using static WCA and HDCA. In addition, a pure PET film was also prepared and annealed at the same conditions to identify its wettability with water and oil. The wettability results are displayed in Table 1. It is obvious that the pure PET film is nearly completely wettable with hexadecane (HDCA < 5°) and partially wettable with water (WCA ≈ 58°). We found that the addition of 5% of FOPU to the PET significantly increases both HDCA and WCA. However, the contact angles’ values are slightly lower than the contact angles measured for the films made from pure FOPUs. This result indicates that the wetting liquids penetrate into/through the polyurethane layer and contact (FOPU/PET) moieties having higher surface energy than CF_3_ and CF_2_ groups. It is noticeable that the surface wettability of the blended films depends on the chemical structure of the fluorinated polyurethane oligomers. For instance, the HDCA of FOPU/PET films without C_4_F_9_-PFPE- end-segments (HFOPU-1 and MFOPU-1) was on the level of 53–55° and WCA on the level of 90–100°. When HFOPU-2 with aliphatic urethane segments and C_4_F_9_-PFPE-end-groups was added to PET, the HDCA of the films increased from 0–5° (HDCA of PET) to 67°, and WCA was increased from 58° (WCA of PET) to 88°. The highest HDCA and WCA were about 72° and 116°, respectively, and were reached with the addition of MFOPU-2, which possesses aromatic urethane linkage and C_4_F_9_-PFPE-end-segments in the oligomeric chains. The oil repellency of MFOPU/PET film is higher than that of PTFE (HDCA = 51°), while the film’s water repellency is practically the same as the repellency of PTFE (WCA = 118°). 

In addition to CA measurements, the surface energy was also estimated to characterize further the surface properties of the FOPU blended films. First of all, the values were calculated from HDCA and WCA data using the Owens–Wendt method (SI: S7). Table 1 shows that pure PET films possess relatively high surface energy around 46 mN/m. A major surface energy decrease is observed for 5 wt% FOPU modified PET films. The surface energy for the films is about 22.3 and 21.3 mN/m for HFOPU-1 and HFOPU-2, respectively. For the PET films blended with MFOPU-1, the surface energy (~19.2 mN/m) is approaching the PTFE level (~17.5 mN/m). As can be anticipated from CA data, the MFOPU-2/PET system has a lower surface energy (~12 mN/m) than that of PTFE. In addition, we also determine the surface energy from HDCA using Figure 2a as a calibration graph (Table 1). The comparison between the surface energy values obtained by the two different methodologies indicates that rubbery/semicrystalline HFOPU macromolecules allow water molecules to penetrate into/through the polymer layer and contact moieties with higher surface energy. In contrast, glassy/semicrystalline MFOPU macromolecules do not allow for such penetration.

Based on the wettability of the MFOPU-1/PET and HFOPU-1/PET surfaces with hexadecane, we can estimate the fraction of the surface occupied by CF_2_ moieties using Equation (3), where the contact angle for all other than CF_2_ groups is considered to be zero. The calculations show that CF_2_ groups occupy ~95 and 91% of the surface for HFOPU-1/PET and MFOPU-1/PET, respectively. Extending Equation (3) to the three-component surface and assuming that the CF_2_:(other moieties) ratio does not change significantly, we can estimate that CF_3_:CF_2_:(other moieties) ratio is 0.27:0.69:0.04 and 0.41:0.52:0.07 for HFOPU-2/PET and MFOPU-2/PET, respectively. The calculations show that only a very small fraction of the surface is not shielded by the fluorinated moieties and that the surface concentration of CF_3_ groups is practically the same in FOPU/PET films and the films made from the pure polyurethanes.

## 3. Conclusions

In summary, we demonstrate that perfluoropolyether-based polyurethane oligomers, especially the ones terminated with CF_3_ moieties, can be employed as safer replacements to long-chain perfluoroalkyl substances/additives. These materials are synthesized in a single-step procedure from commercially available reactants and possess low levels of water and oil wettability. The FOPU macromolecules, when added to an engineering thermoplastic (PET) film, readily migrate to the film surface and bring significant water and oil repellency to the thermoplastic boundary. The best performing FOPU/PET films reached levels of oil wettability and surface energies significantly lower than that of polytetrafluoroethylene, a fully perfluorinated polymer. Specifically, the highest level of the repellency is observed with MFOPU-2 oligomeric additive, which is made using aromatic diisocyanates as comonomers and having CF_3_ end-groups. This semicrystalline oligomer has a T_g_ well above room temperature, and we associate the superiority of this material in achieving low water and oil wettability with its ability to better retain CF_3_ and CF_2_ moieties in contact with the test wetting liquids.

## Figures and Tables

**Table 1:** Wettability and surface energy of perfluoropolyether (PFPE)-based oligomeric polyurethanes (FOPUs), FOPU/polyethylene terephthalate (PET), PET, and polytetrafluoroethylene (PTFE) films.

FOPU	WCA (Degree)	HDCA (Degree)	σ (mN/m) Owens-Wendt	σ (mN/m) from Figure 2
HFOPU-1	92	58	21	17.8
HFOPU-2	99	68	16.4	15
MFOPU-1	114	65	14	15.9
MFOPU-2	120	75	10.6	13.1
HFOPU-1/PET	90	55	22.3	18.5
HFOPU-2/PET	88	67	21.3	15.2
MFOPU-1/PET	100	53	19.2	19.1
MFOPU-2/PET	116	72	12	13.9
PET	58	0–5	46	N/A
PTFE	118	51	17.5	19.6

## Data Availability

The data presented in this study are available on request from the corresponding author.

## Supplementary Materials

The following are available online at https://www.mdpi.com/article/10.3390/polym13071128/s1, S1: Materials, S2: Synthesis of FOPUs, S3: Characterization of FOPUs, S4: Estimation of FOPU heat of fusion and degree of crystallinity, S5: Polymer film preparation, S6: Calculation of surface and interfacial energy, S7. Estimation of surface energy Owens–Wendt method, S8. References.

## References

[1] Gugliuzza A., Drioli E. (2013). A review on membrane engineering for innovation in wearable fabrics and protective textiles. J. Membr. Sci..

[2] Walters K.B., Schwark D.W., Hirt D.E. (2003). Surface characterization of linear low-density polyethylene films modified with fluorinated additives. Langmuir.

[3] Buck R.C., Franklin J., Berger U., Conder J.M., Cousins I.T., de Voogt P., Jensen A.A., Kannan K., Mabury S.A., van Leeuwen S.P.J. (2011). Perfluoroalkyl and polyfluoroalkyl substances in the environment: Terminology, classification, and origins. Integr. Environ. Assess. Manag..

[4] Chan C.-M. (1994). Polymer Surface Modification and Characterization.

[5] Van Krevelen D.W. (2000). Properties of Polymers: Their Correlation with Chemical Structure; Their Numerical Estimation and Prediction from Additive Group Contributions.

[6] Bicerano J. (2002). Prediction of Polymer Properties.

[7] Sperling L. (2006). Introduction to Physical Polymer Science.

[8] Fox H.W., Zisman W.A. (1950). The spreading of liquids on low energy surfaces. I. polytetrafluoroethylene. J. Colloid Sci..

[9] Hare E.F., Shafrin E.G., Zisman W.A. (1954). Properties of Films of Adsorbed Fluorinated Acids. J. Phys. Chem..

[10] Zisman W.A. (1964). Relation of the Equilibrium Contact Angle to Liquid and Solid Constitution. Contact Angle, Wettability and Adhesion.

[11] Ameduri B., Sawada H. (2017). Fluorinated Polymers: Volume 1: Synthesis, Properties, Processing and Simulation.

[12] Ameduri B., Sawada H. (2017). Fluorinated Polymers: Volume 2: Applications.

[13] Kissa E. (2001). Fluorinated Surfactants and Repellents.

[14] Soto D., Ugur A., Farnham T.A., Gleason K.K., Varanasi K.K. (2018). Short-Fluorinated iCVD Coatings for Nonwetting Fabrics. Adv. Funct. Mater..

[15] https://www.nationalgeographic.com/science/2020/01/pfas-contamination-safe-drinking-water-study/.

[16] Guo J., Resnick P., Efimenko K., Genzer J., DeSimone J.M. (2008). Alternative Fluoropolymers to Avoid the Challenges Associated with Perfluorooctanoic Acid. Ind. Eng. Chem. Res..

[17] Conder J.M., Hoke R.A., De Wolf W., Russell M.H., Buck R.C. (2008). Are PFCAs Bioaccumulative? A Critical Review and Comparison with Regulatory Criteria and Persistent Lipophilic Compounds. Environ. Sci. Technol..

[18] US Environmental Protection Agency: Long-Chain Perfluorinated Chemicals (PFCs) Action Plan. https://www.epa.gov/assessing-and-managing-chemicals-under-tsca/long-chain-perfluorinated-chemicals-pfcs-action-plan.

[19] Camaiti M., Brizi L., Bortolotti V., Papacchini A., Salvini A., Fantazzini P. (2017). An environmental friendly fluorinated Oligoamide for producing nonwetting coatings with high performance on porous surfaces. ACS Appl. Mater. Interfaces.

[20] Wei L., Demir T., Grant A.M., Tsukruk V.V., Brown P.J., Luzinov I. (2018). Attainment of water and oil repellency for engineering thermoplastics without long-chain perfluoroalkyls: Perfluoropolyether-based triblock polyester additives. Langmuir.

[21] Demir T., Wei L., Nitta N., Yushin G., Brown P.J., Luzinov I. (2017). Toward a Long-Chain Perfluoroalkyl Replacement: Water and Oil Repellency of Polyethylene Terephthalate (PET) Films Modified with Perfluoropolyether-Based Polyesters. ACS Appl. Mater. Interfaces.

[22] Toselli M., Messori M., Bongiovanni R., Malucelli G., Priola A., Pilati F., Tonelli C. (2001). Poly(ϵ-caprolactone)-poly(fluoroalkylene oxide)-poly(ϵ-caprolactone) block copolymers. 2. Thermal and surface properties. Polymers.

[23] Jellali R., Paullier P., Fleury M.-J., Leclerc E. (2016). Liver and kidney cells cultures in a new perfluoropolyether biochip. Sens. Actuators B Chem..

[24] Lopez G., Ameduri B., Habas J.-P. (2016). A Versatile Strategy to Synthesize Perfluoropolyether-Based Thermoplastic Fluoropolymers by Alkyne-Azide Step-Growth Polymerization. Macromol. Rapid Commun..

[25] Toselli M., Pilati F., Fusari M., Tonelli C., Castiglioni C. (1994). Fluorinated poly(butylene terephthalate): Preparation and properties. J. Appl. Polym. Sci..

[26] Credi C., Levi M., Turri S., Simeone G. (2017). Stereolithography of perfluoropolyethers for the microfabrication of robust omniphobic surfaces. Appl. Surf. Sci..

[27] Fabbri E., Fabbri P., Messori M., Pilati F., Tonelli C., Toselli M. (2004). Surface modification of unsaturated polyester resins with perfluoropolyethers. Polimery.

[28] Pilati F., Toselli M., Vallieri A., Tonelli C. (1992). Synthesis of polyesters-perfluoropolyethers block copolymers. Polym. Bull..

[29] Turri S., Barchiesi E., Levi M. (1995). NMR of perfluoropolyether diols and their acetal copolymers. Macromolecules.

[30] Turri S., Radice S., Canteri R., Speranza G., Anderle M. (2000). Surface study of perfluoropolyether-urethane cross-linked polymers. Surf. Interface Anal..

[31] Drysdale N.E., Brun Y., Mccord E.F., Nederberg F. (2012). Melt Derived Blocky Copolyesters: New Design Features for Polycondensation. Macromolecules.

[32] Vaidya A., Chaudhury M.K. (2002). Synthesis and Surface Properties of Environmentally Responsive Segmented Polyurethanes. J. Colloid Interface Sci..

[33] Wang Z., Macosko C.W., Bates F.S. (2015). Fluorine-Enriched Melt-Blown Fibers from Polymer Blends of Poly(butylene terephthalate) and a Fluorinated Multiblock Copolyester. ACS Appl. Mater. Interfaces.

[34] Valsecchi R., Turri S., Tonelli C., Meroni G., Metta M. (2007). Surface properties modification of thermoplastic polymers by compounding with perfluoropolyether additives. Chim. Oggi Chem. Today.

[35] Bongiovanni R., Malucelli G., Lombardi V., Priola A., Siracusa V., Tonelli C., Di Meo A. (2001). Surface properties of methacrylic copolymers containing a perfluoropolyether structure. Polymers.

[36] Wei L., Caliskan T.D., Tu S., Choudhury C.K., Kuksenok O., Luzinov I. (2020). Highly Oil-Repellent Thermoplastic Boundaries via Surface Delivery of CF3 Groups by Molecular Bottlebrush Additives. ACS Appl. Mater. Interfaces.

[37] Caliskan T.D., Luzinov I. (2020). Effect of number of –CF3 groups in tails of polyester on surface wettability of coatings: Synthesis and characterization of PFPE based polyesters with three -CF3 groups in tails. J. Polym. Res..

[38] Smirnova O., Glazkov A., Yarosh A., Sakharov A. (2016). Fluorinated Polyurethanes, Synthesis and Properties. Molecules.

[39] Wang P.-C., Lu D., Wang H., Bai R.-K. (2019). A New Strategy for the Synthesis of Fluorinated Polyurethane. Polymers.

[40] Zhu Q., Han C.C. (2010). Study of telechelic polyurethane with perfluoropolyether tails. Polymers.

[41] Khayet M., García-Payo C., Matsuura T. (2019). Superhydrophobic nanofibers electrospun by surface segregating fluorinated amphiphilic additive for membrane distillation. J. Membr. Sci..

[42] Game P., Sage D., Chapel J.-P. (2002). Surface Mobility of Polyurethane Networks Containing Fluorinated Amphiphilic Reactive Additives. Macromolecules.

[43] Ho T., Wynne K.J. (1992). A new fluorinated polyurethane: Polymerization, characterization, and mechanical properties. Macromolecules.

[44] Manvi G.N., Singh A.R., Jagtap R.N., Kothari D. (2012). Isocyanurate based fluorinated polyurethane dispersion for anti-graffiti coatings. Prog. Org. Coat..

[45] Jiang G., Tuo X., Wang D., Li Q. (2009). Preparation, characterization, and properties of fluorinated polyurethanes. J. Polym. Sci. Part A Polym. Chem..

[46] Ge Z., Zhang X.Y., Dai J.B., Li W.H., Luo Y.J. (2008). Synthesis and characterization of fluorinated polyurethane with fluorine-containing pendent groups. Chin. Chem. Lett..

[47] Zhao M., Li H., Wen L., Yu Z., Zhang S., Han Z. (2016). Synthesis and characterization of fluorine-containing polyurethane-acrylate core-shell emulsion. J. Appl. Polym. Sci..

[48] Chen K.-Y., Kuo J.-F. (2000). Synthesis and properties of novel fluorinated aliphatic polyurethanes with fluoro chain extenders. Macromol. Chem. Phys..

[49] Ge Z., Zhang X., Dai J., Li W., Luo Y. (2009). Synthesis, characterization and properties of a novel fluorinated polyurethane. Eur. Polym. J..

[50] Yoon S.C., Ratner B.D. (1988). Surface and bulk structure of segmented poly(ether urethanes) with perfluoro chain extenders. 3. Effects of annealing, casting solvent, and casting conditions. Macromolecules.

[51] Tonelli C., Trombetta T., Scicchitano M., Simeone G., Ajroldi G. (1996). New fluorinated thermoplastic elastomers. J. Appl. Polym. Sci..

[52] Tonelli C., Ajroldi G. (2003). New fluoro-modified thermoplastic polyurethanes. J. Appl. Polym. Sci..

[53] Yoon S.C., Ratner B.D. (1988). Surface and bulk structure of segmented poly (ether urethanes) with perfluoro chain extenders. 2. FTIR, DSC, and X-ray photoelectron spectroscopic studies. Macromolecules.

[54] Yang W., Cheng X., Wang H., Liu Y., Du Z. (2017). Surface and mechanical properties of waterborne polyurethane films reinforced by hydroxyl-terminated poly(fluoroalkyl methacrylates). Polymers.

[55] Liu T., Ye L. (2010). Synthesis and properties of fluorinated thermoplastic polyurethane elastomer. J. Fluor. Chem..

[56] Wu D., Ming W., van Benthem R.A.T.M., de With G.B. (2008). Superhydrophobic Fluorinated Polyurethane Films. J. Adhes. Sci. Technol..

[57] Wang X., Hu J., Li Y., Zhang J., Ding Y. (2015). The surface properties and corrosion resistance of fluorinated polyurethane coatings. J. Fluor. Chem..

[58] Bernardini J., Licursi D., Anguillesi I., Cinelli P., Coltelli M.-B., Antonetti C., Galletti A.M.R., Lazzeri A. (2017). Exploitation of Arundo donax L. Hydrolysis Residue for the Green Synthesis of Flexible Polyurethane Foams. Bioresources.

[59] Delebecq E., Pascault J.-P., Boutevin B., Ganachaud F. (2013). On the Versatility of Urethane/Urea Bonds: Reversibility, Blocked Isocyanate, and Non-isocyanate Polyurethane. Chem. Rev..

[60] Hiemenz P.C., Lodge T.P. (2007). Polymer Chemistry.

[61] Fried J.R. (2003). Polymer Science and Technology.

[62] Owens D.K., Wendt R.C. (1969). Estimation of the surface free energy of polymers. J. Appl. Polym. Sci..

[63] Sullivan D.E. (1981). Surface tension and contact angle of a liquid–solid interface. J. Chem. Phys..

[64] Zhang J., Li J., Han Y. (2004). Superhydrophobic PTFE Surfaces by Extension. Macromol. Rapid Commun..

[65] Jańczuk B., Wójcik W., Zdziennicka A., Bruque J.M. (1996). Components of the surface free energy of low rank coals in the presence of n-alkanes. Powder Technol..

[66] Tavana H., Jehnichen D., Grundke K., Hair M.L., Neumann A.W. (2007). Contact angle hysteresis on fluoropolymer surfaces. Adv. Colloid Interface Sci..

[67] Tavana H., Lam C.N.C., Grundke K., Friedel P., Kwok D.Y., Hair M.L., Neumann A.W. (2004). Contact angle measurements with liquids consisting of bulky molecules. J. Colloid Interface Sci..

[68] Luzinov I., Xi K., Pagnoulle C., Huynh-Ba G., Jérôme R. (1999). Composition effect on the core–shell morphology and mechanical properties of ternary polystyrene/styrene–butadiene rubber/polyethylene blends. Polymers.

[69] Wu S. (1971). Calculation of interfacial tension in polymer systems. J. Polym. Sci. Part C Polym. Symp..

[70] Shafrin E.G., Zisman W.A. (1964). Upper limits for the contact angles of liquids on solids. Contact Angle, Wettability, and Adhesion.

[71] Israelachvili J.N., Gee M.L. (1989). Contact angles on chemically heterogeneous surfaces. Langmuir.

[72] Lee H.-H., Gavutis M., Ruželė Ž., Valiokas R., Liedberg B. (2018). Mixed Self-Assembled Monolayers with Terminal Deuterated Anchors: Characterization and Probing of Model Lipid Membrane Formation. J. Phys. Chem. B.

[73] Lilge I., Schönherr H. (2016). Control of Cell Attachment and Spreading on Poly(acrylamide) Brushes with Varied Grafting Density. Langmuir.

[74] Luzinov I., Julthongpiput D., Tsukruk V.V. (2000). Thermoplastic elastomer monolayers grafted to a functionalized silicon surface. Macromolecules.

