# Supplementary material for: Towards a Long-Chain Perfluoroalkyl Replacement: Water and Oil Repellent Perfluoropolyether-Based Polyurethane Oligomers
Source: Polymers (Basel). 2021 Apr 2;13(7):1128. doi: 10.3390/polym13071128 (PMC8036271; doi:10.3390/polym13071128)
Supplement: Supplementary file 1 [file polymers-13-01128-s001.pdf]

SUPPORTING INFORMATION FOR

Towards a Long-Chain Perfluoroalkyl Replacement: Water and Oil Repellent Perfluoropolyether-Based Polyurethane Oligomers

*Liying Wei[1], Tugba D. Caliskan [1,2], Philip J. Brown[1], Igor Luzinov[1]**

[1]Department of Materials Science and Engineering, Clemson University, Clemson, South Carolina 29634, USA

[2]Department of Chemical Engineering, Faculty of Engineering, Ankara University, Tandogan 06100, Ankara, Turkey

*To whom correspondence should be addressed: e-mail: luzinov@clemson.edu

S1. Materials.

S2. Synthesis of FOPUs.

S3. Characterization of FOPUs.

S4. Estimation of FOPU heat of fusion and degree of crystallinity.

S5. Polymer film preparation.

S6. Calculation of surface and interfacial energy.

S7. Estimation of surface energy Owens-Wendt method.

S8. References.

S1. Materials

Perfluoropolyether (PFPE)-based oligomeric polyurethanes (FOPUs) were synthesized using isocyanates from Sigma-Aldrich (1, 6 hexamethylene diisocyanate (HDI) and 4, 4′-methylenebis(phenyl isocyanate) (MDI), fluorinated ether alcohol(s) from Synquest Laboratories (1H, 1H, 11H, 11H- fluorinated-3,6,9-trioxaundecane-1,11-diol (PFPE-diol) and 1H, 1H-fluorinated-3,6,9-trioxatridecan-1-ol (C_4F_9-PFPE-OH)). Methyl ethyl ketone (MEK), purchased from Alfa Aesar, was dried by molecular sieves under vacuum and used as a solvent. Dibutyltin dilautrate (DBTDL) from Sigma-Aldrich was used as a catalyst during synthesis. For the polymer film fabrication, 1,1,1,3,3,3-hexafluoro-2-propanol (HFIP) from Oakwood Products, Inc. was used as a solvent. Commercial grade PET pellets from Unifi were used as received.

S2. Synthesis of FOPUs

The synthesis of FOPU oligomers was conducted via step growth polymerization in solution. The general scheme of reaction is presented in **Figure S1**. To obtain FOPU oligomer with different chemical structures, the synthesis was divided into two groups. HDI or MDI in MEK solution was added dropwise to the solution of (i) PFPE-diol alcohol in dry MEK (HFOPU-1 and MFOPU-1), and (ii) PFPE-diol and C_4F_9-PFPE-OH alcohol solution in MEK (HFOPU-2 and MFOPU-2). The obtained solution was placed in a 100 mL three-necked flask, which was equipped with a mechanical stirrer. DBTDL was used as a catalyst and was added to the reaction solution at room temperature. Subsequently, the solution was heated to 75°C and kept at this temperature for 2h with vigorous stirring under nitrogen stream. The concentration of the catalyst was 0.2-0.3% by weight of the reactants, and the concentration of the reactants in the solution was 30-35% (w/v).

To obtain HFOPU-1, 1.8 g (4.39 mmol) of PFPE-diol was dissolved in 3 ml MEK. Then, a solution of 0.74 g (4.39 mmol) of HDI in dry MEK (5 ml) was added drop-wise into the PFPE-diol solution. The reaction was conducted following the above-written procedure. The final product was dried with N_2. A lightly yellow HFOPU-1 oligomer was obtained.

For the synthesis of MFOPU-1 oligomer, 1.8 g (4.39 mmol) of PFPE-diol was dissolved in 3 ml MEK. Then, a solution of 1.10 g (4.39 mmol) of MDI in dry MEK (5 ml) was added to PFPE-diol solution dropwise. Consequently, the reaction was carried out following the above-written procedure. The final product was dried with N_2. A lightly yellow MFOPU-1 oligomer was obtained.

To obtain HFOPU-2 oligomer, a solution of 3.6 g (8.78 mmol) of PFPE-diol and 1.07 g (1.95 mmol) of C_4F_9-PFPE-OH were dissolved in 10 ml MEK. Then, 1.8 g (4.39 mmol) of PFPE-diol was dissolved in 3 ml MEK. Subsequently, 1.64 g (9.75 mmol) of HDI in dry MEK (10 ml) was added dropwise into the PFPE-based alcohols solution to prepare HFOPU-2 using procedure described above. After the drying, a lightly yellow HFOPU-2 was obtained.

To obtain MFOPU-2 oligomer, 2.44 g (9.75 mmol) of MDI in 10 ml dry MEK was added dropwise into a solution of 3.6 g (8.78 mmol) of PFPE-diol and 1.07 g (1.95 mmol) of C_4F_9-PFPE-OH in 10 ml MEK. Then, the reaction was conducted following the procedure above. After the drying, a lightly yellow MFOPU-2 oligomer was obtained.

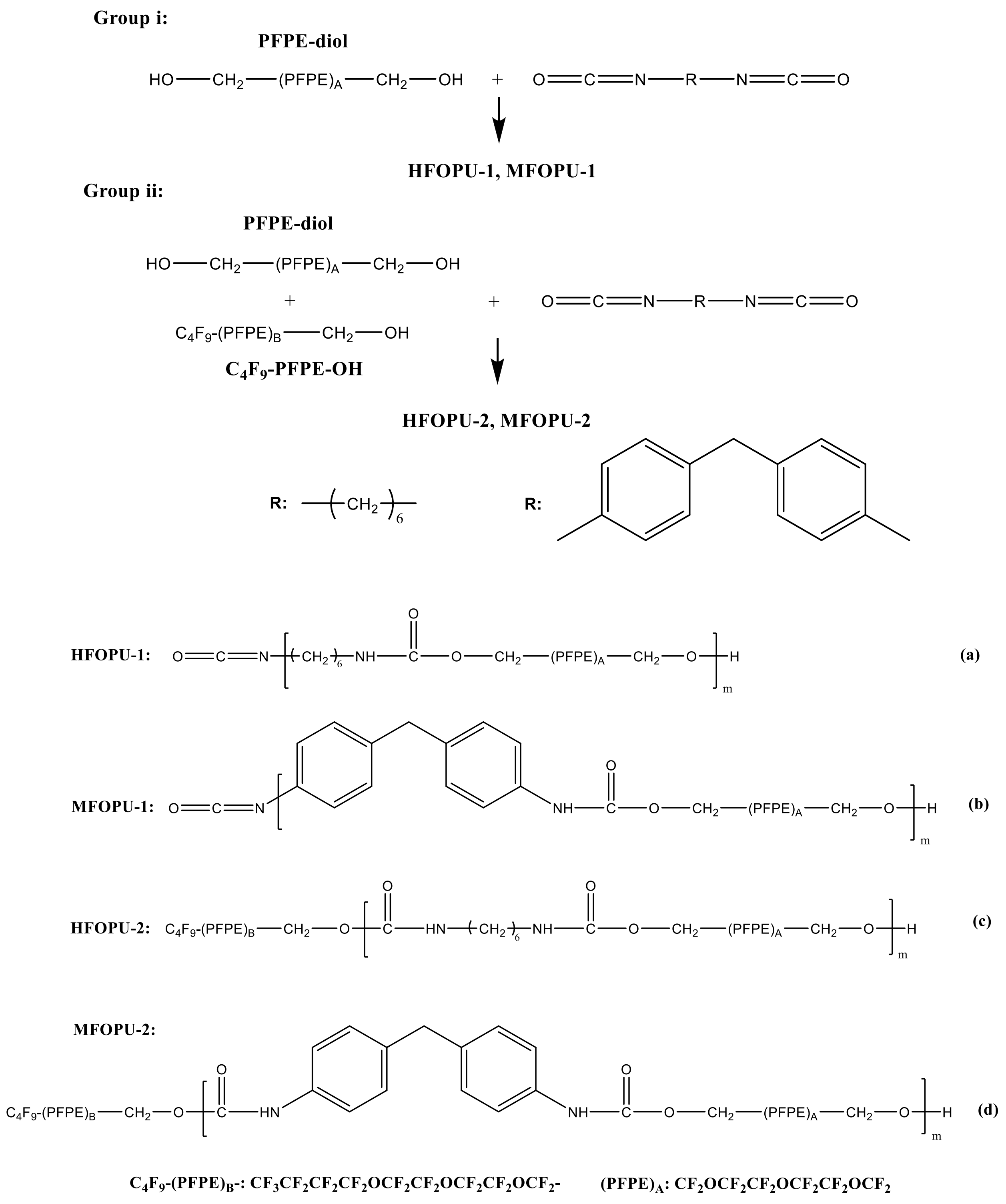

Figure S1. General schematics for synthesis of FOPUs. Chemical structure of (a) HFOPU-1 polyurethane, (b) MFOPU-1 polyurethane, (c) HFOPU-2 polyurethane, and (d) MFOPU-2 polyurethane.

S3. Characterization of FOPUs

Attenuated Total Reflectance Fourier Transform Infrared Spectroscopy (ATR-FTIR) spectra of the materials were acquired with a Thermo Nicolet 6700 FTIR spectrometer. For fluorine nuclear magnetic resonance spectroscopy (^{19}F NMR), the dried materials were dissolved in deuterated chloroform (with trichlorofluoromethane as a reference) for 24h. Bruker Avance II Spectrometer (300 MHz) was used to record the ^{19}F NMR for the samples.

The molecular weight of the materials synthesized in this work were measured by gel permeation chromatography (GPC Waters Breeze). Prior to the measurements, the oligomers were dissolved in chloroform and kept overnight. The obtained oligomer solutions were filtered through the 0.2μm syringe PTFE filters. Polystyrene was employed as a standard for the GPC calibration.

For thermogravimetric analysis (TGA), Perkin Elmer TGA was used. Samples approximately 5 mg were pre-treated under a nitrogen atmosphere (gas flow of 20 mL/min) for 10 min at 25°C. Then samples were heated to 600°C at a heating rate of 20°C/min under N_2. For differential scanning calorimetry (DSC), a DSC 2920 (TA Instruments) was employed. Samples (~ 5 mg) were analyzed at a heating rate of 20°C/min under nitrogen atmosphere.

Atomic force microscopy (AFM, DI 3100 Dimensions) was conducted with a Dimension 3100 microscope (Digital Instruments, Inc.) in order to scan polymer films in tapping mode at 1Hz scan rate. The static contact angles of water (WCA) and hexadecane (HDCA) were measured at room temperature with an equilibration time of 30 sec. CAs were recorded with drop shape analysis instrument (DSA10, Kruss, Germany).

ATR-FTIR spectroscopy

The major functional groups presented in the obtained FOPUs were identified using ATR-FTIR analysis. The IR spectra are displayed in **Figure S2** and they were analyzed using readily available spectra databases for organic compounds [1]. In general, the IR results **(Table S1)** indicated that FOPUs were obtained by the synthetic procedure employed. IR spectra for all four FOPUs possessed the -NH stretching and -NH bending peaks around 3340 cm^{-1} and 1535 cm^{-1}, respectively. These peaks were formed as a result of isocyanate reaction with alcohol. Furthermore, -OC=O ester stretching peaks around 1700 cm^{-1} and -C-O-C- stretching vibration peaks at 1283 cm^{-1} were also identified. All four oligomers possessed -CF_2 and -CF_3 stretching vibrations in the region of 1200-1100 cm^{-1}. For HFOPU oligomers, the -CH aliphatic stretching peaks were detected in the region of 2938-2861 cm^{-1}. On the other hand, the -CH aromatic stretching peaks were found between 3100 cm^{-1} and 3000 cm^{-1} for MFOPU oligomers. In addition, the -CH_2 scissoring peak at 1415 cm^{-1} in the MFOPUs was much stronger than it in HFOPUs because of the connection of -CH_2 group with the two aromatic rings in MDI monomers.

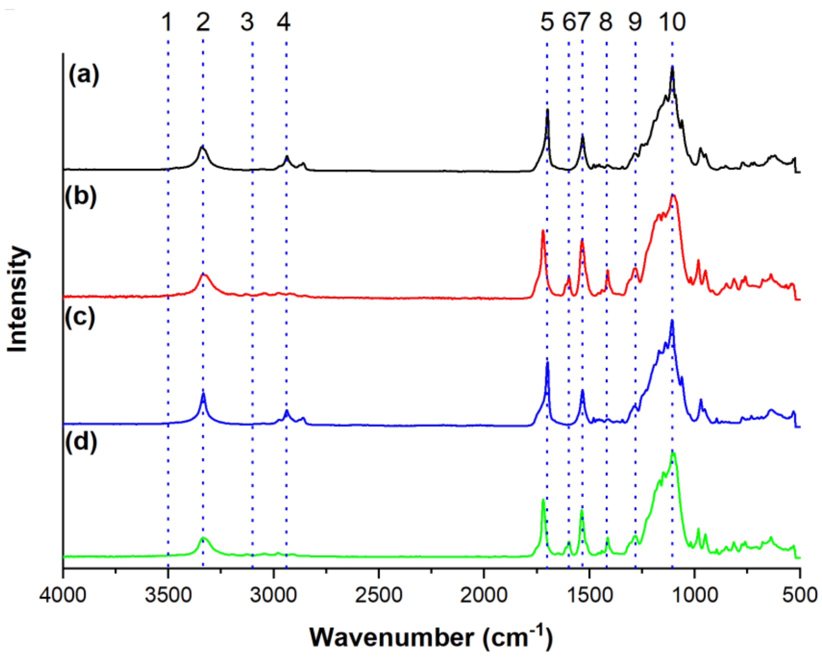

Figure S2. ATR-FTIR spectra of FOPUs: (a) HFOPU-1, (b) MFOPU-1, (c) HFOPU-2, and (d) MFOPU-2. Mark on spectra: (1) -OH stretching, 3500-3450 cm^{-1}, (2) -NH stretching, 3335 cm^{-1}, (3) -CH stretching (aromatic), 3100-3000 cm^{-1}, (4) -CH stretching (aliphatic), 2938-2861 cm^{-1}, (5) -OC=O stretching, 1701 cm^{-1}, (6) -C=C- stretching (aromatic), 1598 cm^{-1}, (7) -NH bending, 1533 cm^{-1}, (8) -CH_2 scissoring, 1283 cm^{-1}, (9) -C-O-C symmetric stretching, 1283 cm^{-1}, (10) -CF_2 and -CF_3 stretching,1139-100 cm^{-1}.

Table S1. IR absorption bands of FOPUs.

Absorbing group and type of vibration	HFOPU-1 wavenumber (cm^{-1})	MFOPU-1 wavenumber (cm^{-1})	HFOPU-2 wavenumber (cm^{-1})	MFOPU-2 wavenumber (cm^{-1})
-OH stretching	-	-	-	-
-NH stretching	3344	3336	3335	3334
-CH aliphatic stretching	2938-2861	-	2938-2861	-
-CH aromatic stretching	-	3100-3000	-	3100-3000
-OC=O stretching	1701	1721	1701	1721
C=C aromatic stretching	-	1598	-	1598
-NH bending	1533	1537	1533	1538
-CH_2 scissoring	1415 (weak)	1415 (strong)	1415 (weak)	1415 (strong)
-C-O-C- stretching	1283	1283	1283	1283
-CF_2 and -CF_3 stretching	1139-100	1167-100	1140-100	1169-100

NMR spectroscopy

^{19}F NMR analysis was conducted to further investigate the structure of FOPUs. However, the MFOPU oligomers could not be fully dissolved in the deuterated solvents available to us. Therefore, only NMR result for HFOPU-1 and HFOPU-2 is presented in **Figure S3** and **Figure S4**, respectively. Overall, NMR data confirmed the synthesis of targeted FOPUs. For both HFOPU-1 and HFOPU-2 oligomers, the signals at -77.40 to -77.87 ppm (a) are present, which are attributed to the fluorine atom in the CF_2 groups bonded to methyl ester ($-O-\underline{CF_2}-CH_2-O-CO-$) in repeat units. The distinctive multiple peaks at -88.76-89.58 ppm (b) correspond to the fluorine atoms of the $-CF_2-$ groups located between ethers ($-O-\underline{CF_2CF_2}-O-$) in the repeating units. Furthermore, the triplet peaks at -80.38 to -80.55 ppm (f) belong to the fluorine atom in the CF_2 group, which is close to the -OH end groups ($-O-\underline{CF_2}-CH_2-OH$) [2,3]. It confirms that both HFOPU oligomers possessed -OH end-groups. Furthermore, for HFOPU-2 oligomer (**Figure S4**), three additional peaks (c, d, and e) that belong to the fluorine atoms in C_4F_9-PFPE- end segment are detected. The two singlet peaks at -81.09 ppm (c) and -83.57 ppm (d) are attributed to the fluorine atoms in the $-CF_3$ group and $-CF_2$ group bonded to ether ($CF_3-CF_2CF_2-\underline{CF_2}-O$) [4]. Another peak "e" at -126.67 belongs to the fluorine atoms of the $-CF_2$ groups ($CF_3-\underline{CF_2CF_2}-CF_2-O$) on the tail [4]. The results revealed that HFOPU-2 oligomer was terminated with C_4F_9-PFPE- end-groups on one side.

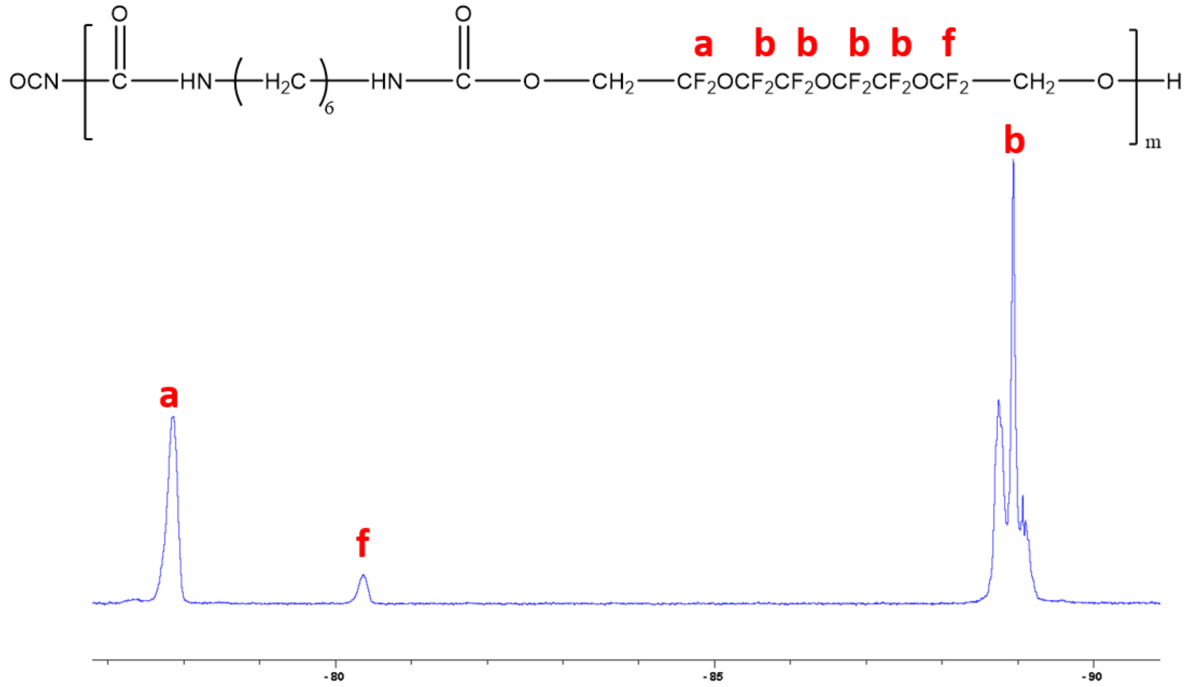

Figure S3. ^{19}F NMR spectrum of HFOPU-1.

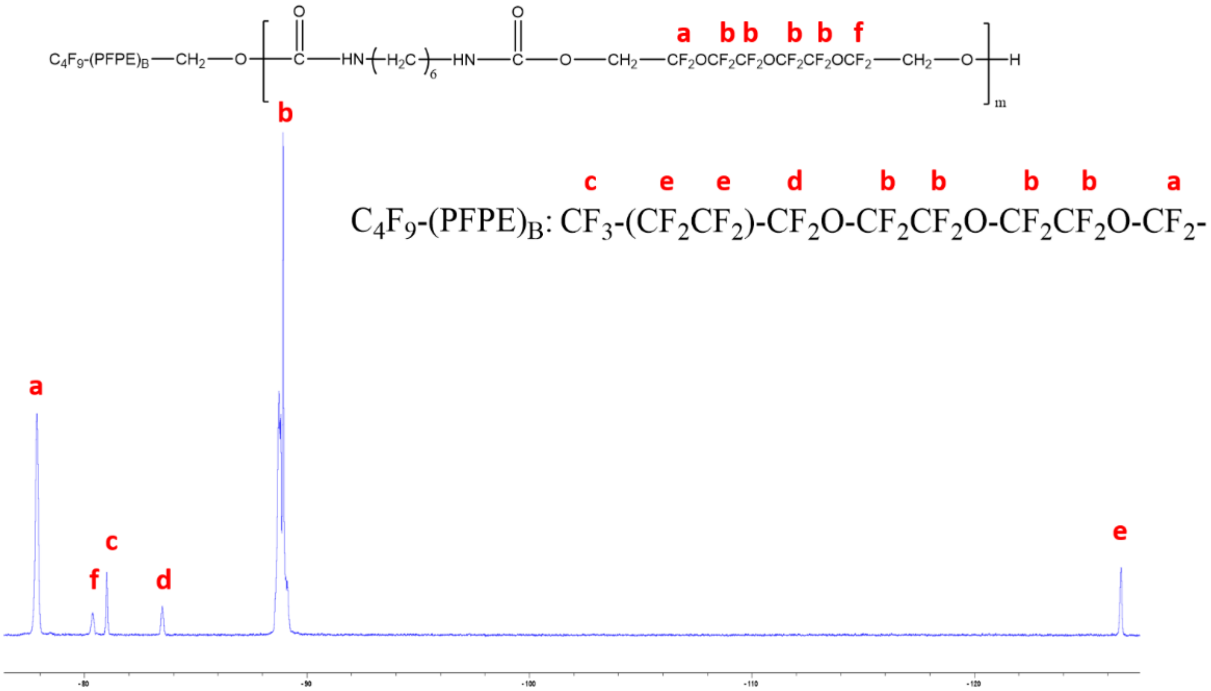

Figure S4. ^{19}F NMR spectrum of HFOPU-2.

GPC Analysis

The weight average molecular weight (M_w), number average molecular weight (M_n) and polydispersity index (PDI) for FOPUs were determined by GPC analysis. Chloroform and polystyrenes was used as solvent and calibration standards for the samples, respectively. HFOPU oligomers can be completely dissolved in chloroform, while MFOPUs can only be partially dissolved. The data obtained from GPC analysis in **Table S2** revealed that HFOPU oligomers with M_w between 2800-4010 g/mol and PDI around ~1.8 were obtained. On the other hand, dissolved fraction of MFOPU oligomers possessed M_w and PDI around 2500 g/mol and 1.2, respectively.

Table S2. Major characteristics of FOPUs.

Oligomer	M_n (g/mol)	M_w (g/mol)	PDI	T_g (ºC)	T_m (ºC)	Crystallinity (%)
HFOPU-1	1588	2879	1.67	-32	60	27.8
MFOPU-1	2320*	2682*	1.16*	45	125	35.6
HFOPU-2	2257	4009	1.78	-28	64	31.1
MFOPU-2	2258*	2556*	1.13*	47	123	34.7

*: molecular weight for oligomer that can be dissolved in chloroform

TGA Analysis

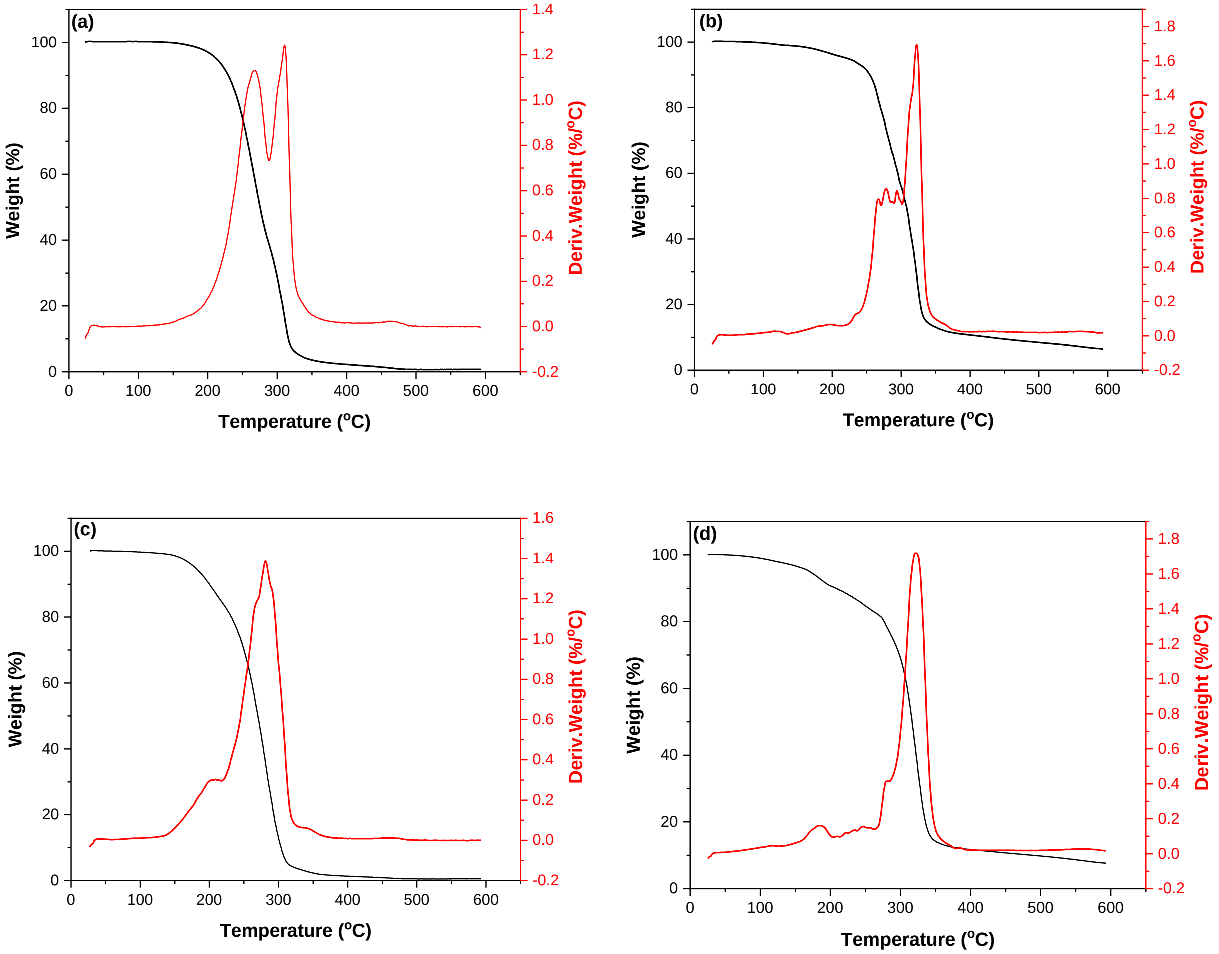

Figure S5. TGA and derivative TGA (DTGA) traces for: (a) HFOPU-1, (b) MFOPU-1, (c) HFOPU-2, and (d) MFOPU-2.

DSC Analysis

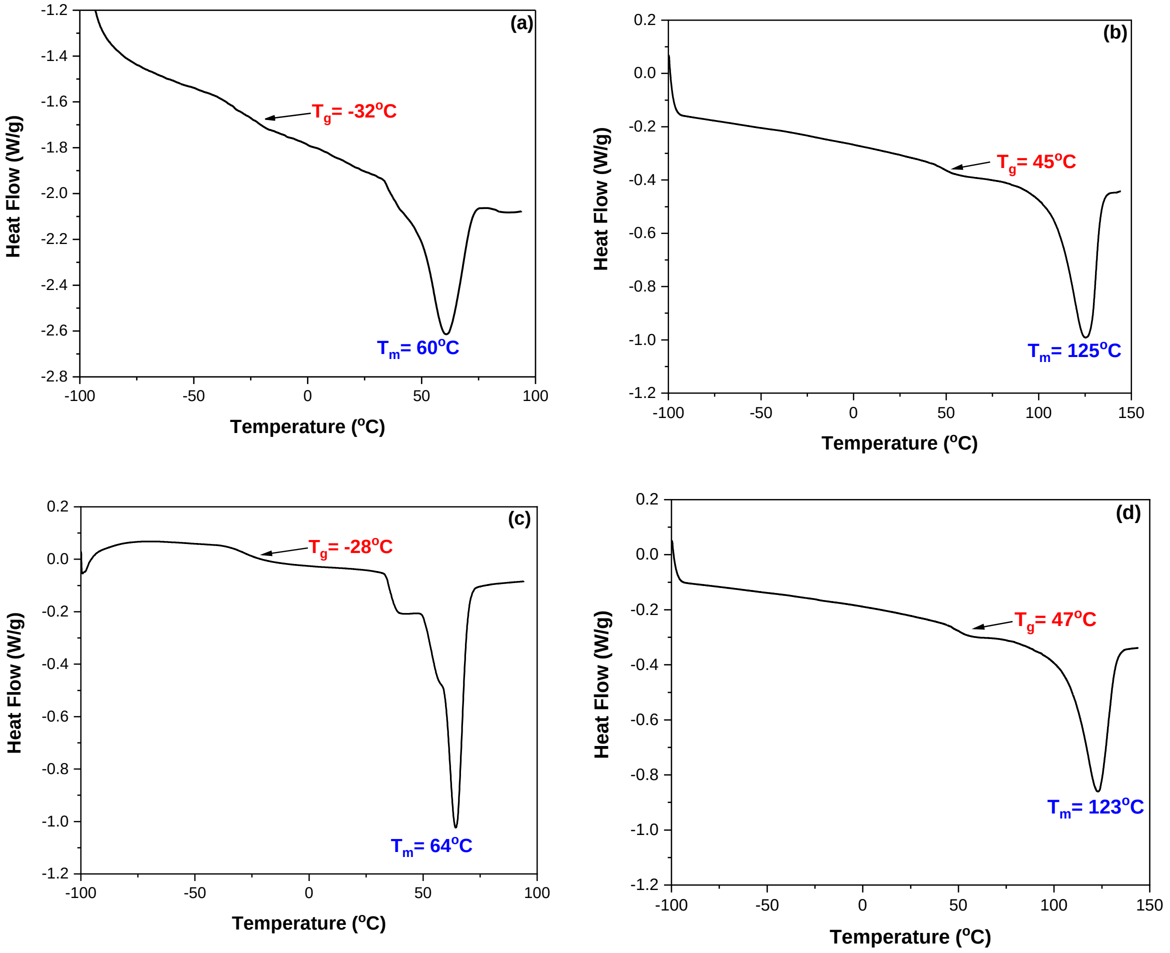

Figure S6. DSC traces for: (a) HFOPU-1, (b) MFOPU-1, (c) HFOPU-2, and (d) MFOPU-2.

S4. Estimation of FOPU heat of fusion and degree of crystallinity

The degree (percentage) of crystallinity for FOPUs was roughly estimated. The percentage was calculated based on the heat of fusion (*ΔH$_f$*) by following equation:

$$\% crystallinity = 100[\frac{\Delta H_f - \Delta H_c}{\Delta H_{crys}^f}] \qquad \textbf{(Eq. S1)}$$

The heat of fusion (*ΔH$_f$*) for FOPUs was obtained from DSC results (**Figure S6)**. Neither of the oligomer has the heat of the additional crystallization (*ΔH$_c$*); hence; the *ΔH$_c$* was considered as 0 J/g. Furthermore, the heat of fusion for 100% crystalline material (ΔH_{crys}^f) (**Table S3**) for FOPUs was determined from the tabulated molar contributions of the chemical groups constituting repeating units according to the method published elsewhere [5]. The obtained degree of crystallinity for the materials is reported in **Table S2**.

Table S3. Tentative values of group contributions to the heat of fusion for FOPUs.

**HFOPU-1**	**MFOPU-1**	**HFOPU-2**	**MFOPU-2**
8 CH_2 = 8x4 = 32 kJ/mol	3 CH_2 = 3x4 = 12 kJ/mol	8 CH_2 = 8x4 = 32 kJ/mol	3 CH_2 = 3x4 = 12 kJ/mol
6 CF_2 = 6x4 = 24 kJ/mol	6 CF_2 = 6x4 = 24 kJ/mol	6 CF_2 = 6x4 = 24 kJ/mol	6 CF_2 = 6x4 = 24 kJ/mol
5 O = 5x1 = 5 kJ/mol	5 O = 5x1 = 5 kJ/mol	5 O = 5x1 = 5 kJ/mol	5 O = 5x1 = 5 kJ/mol
1 CONH = 1x2 = 2 kJ/mol	1 CONH = 1x2 = 2 kJ/mol	2 CONH = 2x2 = 4 kJ/mol	2 CONH = 2x2 = 4 kJ/mol
-	2 -C_6H_4- =2x5 = 10 kJ/mol	-	2 -C_6H_4- =2x5 = 10 kJ/mol
**Total = 63 kJ/mol**	**Total = 53 kJ/mol**	**Total = 65 kJ/mol**	**Total = 55 kJ/mol**
**MW $_{\text{repeat units}}$ = 535 g/mol**	**MW $_{\text{repeat units}}$ = 617 g/mol**	**MW $_{\text{repeat units}}$ = 578 g/mol**	**MW $_{\text{repeat units}}$ = 660 g/mol**
ΔH_{crys}^f **= 117.8 J/g**	ΔH_{crys}^f **= 85.9 J/g**	ΔH_{crys}^f **= 112.5 J/g**	ΔH_{crys}^f **= 83.3 J/g**
ΔH$_f$ **= 32.7 J/g**	***ΔH$_f$*** **= 30.6 J/g**	***ΔH$_f$*** **= 35.0 J/g**	***ΔH$_f$*** **= 28.9 J/g**

S5. Polymer film preparation

To prepare films, FOPU was solvent-blended with PET in HFIP at the concertation of 5%. The blended films were fabricated on clean Si wafer substrates by dip coating (dip coater: Mayer Fientechnik D-3400) from 3 wt% polymer solution in HFIP using 320 mm/min withdrawal rate. Prior to the film deposition, the wafers were first cleaned in an ultrasonic bath for 30 min, placed in a hot "piranha" solution (3:1 concentrated sulfuric acid/ 30% hydrogen peroxide) for 1h, and then rinsed several times with high purity deionized water. After being rinsed, the substrates were dried under a stream of dry nitrogen. After the deposition, the films were kept at room temperature overnight. Afterward, the dried films were annealed at 140°C for 3h in a vacuum oven. Thickness of the polymer films was 300 - 400 nm as measured by ellipsometry with a COMPEL automatic ellipsometer (InOmTech, Inc.) at an angle of 70° and wavelength of 653nm.

S6. Calculation of surface and interfacial energy.

The interfacial tensions between the segments/materials were estimated using the harmonic mean equation (T = 298K) [6,7]:

$$\gamma_{12} = \gamma_1 + \gamma_2 - \frac{4\gamma_1^d \gamma_2^d}{\gamma_1^d + \gamma_2^d} - \frac{4\gamma_1^p \gamma_2^p}{\gamma_1^p + \gamma_2^p} \qquad \textbf{(Eq. S2)}$$

where the *d* and *p* subscripts refer to the dispersive and polar contributions to the surface tensions γ, respectively. For CF_3 and CF_2 groups we considered that the fraction of dispersive contribution to γ is on the same level as the one for PTFE (0.92).

We estimated surface energies and their (polar and dispersive) components for the molecular segments constituting FOPUs (**Figure S6 and Table S4**). The parameters were calculated via Biscerano algorithms [8] (using Polymer Design Tools, Version 1.1, DTW Associates, Inc). **Eq. S3** was utilized to estimate the polar component of the surface tension [6]:

$$\gamma_p / \gamma_T = \left(\delta_p / \delta_T \right)^2 \qquad \textbf{(Eq. S3)}$$

where γ_p and γ_T are the polar component and overall surface tension, respectively and δ_p , δ_t are the polar and total solubility parameters respectively. The dispersive component of the surface tension, γ_d , could then be found utilizing the additive nature of the surface tension relationship:

$$\gamma_T = \gamma_p + \gamma_d \qquad \textbf{(Eq. S4)}$$

The polar component of the solubility parameter for FOPU repeating units, C_4F_9-PFPE- end group, and PET was estimated by first calculating the dispersive component of the solubility parameter, δ_d , from:

$$\delta_d = \frac{F_d}{V_m} \qquad \textbf{(Eq. S5)}$$

where F_d is defined as the dispersion component of the molar attraction [8], and V_m is the molar volume of the monomer unit. From the estimated values of the overall solubility parameter, δ_T, and δ_d, the polar component can be found using the relationship:

$$\delta_T = \sqrt{\delta_d^2 + \delta_p^2} \qquad \textbf{(Eq. S6)}$$

$(PFPE)_A$: – CF_2 – O – CF_2 – CF_2 – O – CF_2 – CF_2 – O – CF_2 –

$(PFPE)_A$ containing segment: – O – CH_2 – $(PFPE)_A$ – CH_2 – O –

HFOPU aliphatic urethane segment:

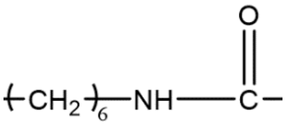

MFOPU aromatic urethane segment:

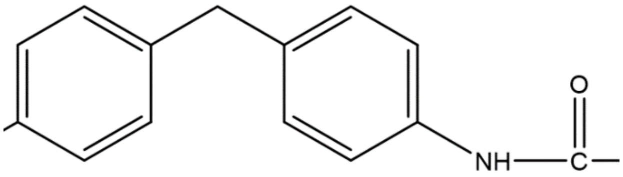

Figure S6. Chemical structures of FOPU molecular segments, which surface energy is reported in Table S4.

Table S4. Surface energies and their (polar and dispersive) components for the molecular segments constituting FOPUs

Segment	γ_T(mN/m)	γ_d(mN/m)	γ_p(mN/m)
HFOPU aliphatic urethane segment	45.8	24.31	21.49
MFOPU aromatic urethane segment	50.8	30.52	20.28
-O-CH_2-$(PFPE)_A$-CH_2-O-	30.8	21.66	9.14
-$(PFPE)_A$-	28.7	20.05	8.65

S7. Estimation of surface energy Owens-Wendt method.

The surface energy of PTFE, PET, FOPU and FOPU/PET films were calculated according to Owens-Wendt method **(Eq. S7)** [9].

$$\gamma_{l1}(1+\cos\theta_1) = 2\sqrt{\gamma_s^d \gamma_{l1}^d} + 2\sqrt{\gamma_s^p \gamma_{l1}^p}$$

$$\gamma_{l2}(1+\cos\theta_2) = 2\sqrt{\gamma_s^d \gamma_{l2}^d} + 2\sqrt{\gamma_s^p \gamma_{l2}^p} \qquad \textbf{(Eq. S7)}$$

$$\gamma_s = \gamma_s^d + \gamma_s^p$$

where γ_s and γ_l are the surface tensions of the solid and liquid, respectively. The subscripts *d* and *p* correspond to dispersion and polar components of the surface tension, respectively. Surface free energy (γ_s) and its polar (γ_s^p) and dispersion (γ_s^d) components of the FOPU/PET surfaces were determined using two sets of contact angle measurements of water and hexadecane. The γ_l^p and γ_l^d components of liquids shown in **Table S5** were used in the calculations.

Table S5. The γ_l^p and γ_l^d components of liquids [10].

	γ_l^d	γ_l^p	γ_l
Hexadecane	26.35	0	26.35
Water	21.8	51	72.8

S8. References

1. Pretsch, E.; Bühlmann, P.; Badertscher, M. IR Spectroscopy. In *Structure Determination of Organic Compounds: Tables of Spectral Data*, Springer Berlin Heidelberg: Berlin, Heidelberg, 2009; 10.1007/978-3-540-93810-1_7pp. 1-67.
2. Wu, J.; Zhou, X.; Harris, F.W. Bis(perfluoro-2-n-propoxyethyl)diacyl peroxide initiated homopolymerization of vinylidene fluoride (VDF) and copolymerization with perfluoro-n-propylvinylether (PPVE). *Polymer* **2014**, *55*, 3557-3563, doi:http://dx.doi.org/10.1016/j.polymer.2014.06.066.
3. Turri, S.; Barchiesi, E.; Levi, M. NMR of perfluoropolyether diols and their acetal copolymers. *Macromolecules* **1995**, *28*, 7271-7275, doi:10.1021/ma00125a034.
4. Karis, T.E.; Marchon, B.; Hopper, D.A.; Siemens, R.L. Perfluoropolyether characterization by nuclear magnetic resonance spectroscopy and gel permeation chromatography. *J. Fluorine Chem.* **2002**, *118*, 81-94, doi:http://dx.doi.org/10.1016/S0022-1139(02)00197-5.

5. Van Krevelen, D.W. *Properties of polymers: their correlation with chemical structure; their numerical estimation and prediction from additive group contributions*, Third ed.; Elsevier: Amsterdam, 2000; pp. 875.
6. Luzinov, I.; Xi, K.; Pagnoulle, C.; Huynh-Ba, G.; Jérôme, R. Composition effect on the core–shell morphology and mechanical properties of ternary polystyrene/styrene–butadiene rubber/polyethylene blends. *Polymer* **1999**, *40*, 2511-2520.
7. Wu, S. Calculation of interfacial tension in polymer systems. *J. Polym. Sci., Part C: Polym. Symp.* **1971**, *34*, 19-30, doi:https://doi.org/10.1002/polc.5070340105.
8. Bicerano, J. *Prediction of polymer properties*; Marcel Dekker: New York-Basel, 2002; pp. 736.
9. Owens, D.K.; Wendt, R.C. Estimation of the surface free energy of polymers. *J. Appl. Polym. Sci.* **1969**, *13*, 1741-1747, doi:10.1002/app.1969.070130815.
10. Jańczuk, B.; Wójcik, W.; Zdziennicka, A.; Bruque, J.M. Components of the surface free energy of low rank coals in the presence of n-alkanes. *Powder Technol.* **1996**, *86*, 229-238.
